# Characterization of Bacterial Communities in Mexican Artisanal Raw Milk “Bola de Ocosingo” Cheese by High-Throughput Sequencing

**DOI:** 10.3389/fmicb.2018.02598

**Published:** 2018-10-29

**Authors:** Alejandro Aldrete-Tapia, Claudia Meyli Escobar-Ramírez, Mark L. Tamplin, Montserrat Hernández-Iturriaga

**Affiliations:** ^1^Departamento de Investigación y Posgrado en Alimentos, Facultad de Química, Universidad Autónoma de Querétaro, Querétaro, Mexico; ^2^Centro Nacional de Investigación Disciplinaria de Fisiología Animal (CENIDFyMA), Instituto Nacional de Investigaciones Forestales, Agrícolas y Pecuarias, Querétaro, Mexico; ^3^Centre of Food Safety & Innovation, Tasmanian Institute of Agriculture, University of Tasmania, Sandy Bay, TAS, Australia

**Keywords:** artisanal cheese, lactic acid bacteria, microbial communities, pyrosequencing, raw milk

## Abstract

The dynamics of bacteria community of “Bola de Ocosingo” cheese, a Mexican artisanal raw milk cheese was investigated by high-throughput sequencing (454 pyrosequencing). Dairy samples (raw milk, curd, cheese at 50 and 110 days of ripening) were collected at dry (March-June) and rainy season (August-November) from three producers located in Chiapas, Mexico. In general, raw milk contained high bacterial diversity which was reduced throughout cheese manufacture. However, in two productions an important increase during cheese ripening was observed probably due to cross-contamination. Species such as *Streptococcus thermophilus*, *Lactococcus lactis*, *Lactobacillus helveticus*, *L. delbrueckii* and *L. plantarum* from which potential probiotic strains may be obtained, predominated during processing, varying its prevalence from one producer to another. Furthermore, low proportions of *Escherichia coli*/*Shigella flexnerii* were detected in almost all processes, however, could not be recovered by traditional methodology, indicating presence of non-cultivable cells. This work provides insights into bacteria communities of Bola de Ocosingo cheese for starter culture development, many of which are reported to provide health related benefits, and the usefulness of high-throughput sequencing to evidence cross-contamination during processing.

## Introduction

“Bola de Ocosingo” cheese is a Mexican artisanal short-ripened cheese elaborated with raw milk produced in Chiapas, Mexico, and is characterized for uneven quality as a result of differences in processing conditions, the use of traditional acidification by autochthonous lactic acid bacteria (LAB) present in the raw materials, and the absence of controls in storage. These factors limit the distribution to regional level and furthermore, represent a health risk for consumers as the raw milk may contain foodborne pathogens (Cervantes et al., 2006).

For cheese-making producers, Mexican normativity indicates the use of pasteurized milk (Nom-243-SSA1-2010, 2010). However, pasteurization eliminates some of the indigenous microflora in the raw milk, affecting adversely sensorial characteristics such as flavor, odor and texture quality (Albenzio et al., 2001). Adding microorganisms (such as LAB) selected from artisanal production process, as starter culture to produce cheese using pasteurized milk would allow the development of desirable sensorial characteristics, constant quality and safety in the final product (Torres-Llanez et al., 2006).

Studies focused on bacterial biodiversity during cheese processing may serve as the first step in the development of starter cultures containing technologically relevant microorganisms from the cheese under study (Riquelme et al., 2015). Furthermore, these microorganisms could provide probiotic effects in the host, if properly isolated and characterized (Monnet and Bogovic, 2012). To address the diversity of microbial communities throughout cheese manufacture and ripening, culture-independent molecular techniques such as pyrosequencing has been applied enabling rapid insight into composition, structure and dynamics of microbial communities (Alegria et al., 2012).

In this study, high-throughput sequencing was used to describe dynamics of bacterial communities during processing of Bola de Ocosingo cheese.

## Materials and Methos

### Cheese

Bola de Ocosingo cheese was made with raw milk from Braunvieh, Brown Swiss and Zebu cows. A portion of the raw milk was skimmed, obtaining cream that was added to the batch process, in a portion of 4 Kg of cream per 100 L of milk. The mixture was curdled using 2.5 mL of commercial calf rennet 1:10,000 (Cuamex, Mexico) for 5–12 h without addition of CaCl_2_. After 4–8 h, the curd was cut, rested for 12 h, transferred to a muslin cloth sack, hanged to drain whey for up to 5 days, and then finally salted (3–4%). Dry curd was transferred to a new muslin sack, hanged for up to 5 days, and this step repeated for 50 days to obtain a ripened curd. Curd was then crumbled and mixed with butter only if needed, in a portion of 0.3–0.5 Kg per 10 Kg of ripened curd, obtaining a texture similar to double-cream cheese, shaped in balls (200–300 g) and covered with *pasta filata* cheese obtained from recently skimmed milk which was acidified with vinegar, curdled, whey was drained, then boiling water was added to melt the curd. It can be sold fresh (50 days of ripening), or aged an additional 2 months (110 days of ripening). All the processing, distribution and storage in the market is at room temperature, the final product is sold without cover other than the hardened *pasta filata*, which is not consumed.

### Sample Collection

Twenty-four samples were obtained from three producers (A, B, and C) in the state of Chiapas, Mexico, collected at dry (March-June) and rainy seasons (August-November). One sample of raw milk, curd, fresh cheese (curd at 50 days of ripening mixed with butter and covered with *pasta filata*) and ripened cheese (110 days of ripening) from the same batch was obtained from each manufacture and season. Samples were stored at -20°C and transported to the Universidad Autónoma de Querétaro for analysis.

### DNA Extraction

Total genomic DNA was extracted as previously reported (Aldrete-Tapia et al., 2014) consisting in a pre-treatment to remove food lipids, proteins and salts, obtaining the cell pellet. Cells were subjected to lysis by heat and powdered glass and DNA was purified with phenol-chloroform, and ethanol precipitation.

### Pyrosequencing (454 Sequencing)

DNA sequencing was performed at MR DNA^1^ (Shallowater, TX, United States). Briefly, the 16S rRNA gene was amplified using the primers 27Fmod (AGRGTTTGATCMTGGCTCAG) and 530R (CCGCNGCNGCTGGCAC); 454-adaptors were included in the forward primer as well as a barcode for each sample. The sequencing was performed utilizing a Roche 454 FLX titanium (Roche diagnostics Ltd., West Sussex, United Kingdom) instrument and reagents, following the manufacturer’s instructions. The sequence data were processed using Mothur version 1.31.2 software (Schloss et al., 2009) with a modified pipeline. Briefly, sequences were subjected to quality controls and the 454-adaptors trimmed; unique sequences were aligned to the SILVA reference database. Chimeras were removed from aligned sequences with the uchime algorithm and classified to obtain the taxonomic assignment using the Silva 16S rRNA gene database. The final sequences were normalized to the lower number of sequences reads (data was subsampled to 1834 sequences). Good’s coverage, Chao1 richness and Inverse Simpson diversity indices were calculated and rarefaction curves produced. Quality sequences were aligned to the 16S rRNA gene sequences using the BLAST tool from NCBI. Permutational multivariate analysis of variance (PERMANOVA) calculating Bray-Curtis dissimilarity was used to determine if proportions of main species detected were different across seasons, producers and sample type with the function *Adonis* in the library *vegan* (Oksanen et al., 2016) for R software (R Core Team, 2016).

## Results

### Bioinformatic Analysis of Sequences

A total of 160,617 quality reads with average length of 280 bp (range = 264 to 327) were obtained from 24 dairy samples after Mothur pipeline. Number of sequence reads and OTUs, as well as diversity, richness and coverage estimators (calculated with an identity at 3% sequence similarity level) are shown in Table 1. Rarefaction curves approached a plateau, meaning most microbial diversity was captured within the number of samples (Supplementary Figure S1). Good’s coverage indicated a satisfactory overall sampling with levels above 99%.

**Table 1:** OTUs identified at 97% similarity, species richness estimate (Chao1), diversity index (inverse Simpson) and coverage for 16S rRNA sequencing of dairy samples from Bola de Ocosingo cheese production.

Manufacturer	Season	Sample	Reads	OTUs	Chao1		Inverse Simpson		Good’s coverage
A	Dry	Milk	7984	217	295.96	(259.19–364.80)	12.97	(12.43–13.56)	99.16%
		Curd	2186	39	73	(49.10–153.42)	3.89	(3.77–4.01)	99.22%
		Cheese 50	2006	30	35	(31.16–51.57)	2.80	(2.69–2.92)	99.5%
		Cheese 110	4014	24	36	(26.66–78.20)	1.12	(1.1–1.13)	99.78%
	Rainy	Milk	12267	311	505.24	(430.29–627.28)	8.22	(7.98–8.49)	99.01%
		Curd	30084	82	122.07	(98.95–176.73)	1.36	(1.35–1.37)	99.89%
		Cheese 50	21104	77	111.36	(90.39–165.16)	2.48	(2.46–2.50)	99.87%
		Cheese 110	19618	162	279.30	(223.46–385.86)	4.22	(4.17–4.27)	99.65%
B	Dry	Milk	3034	19	26	(20.34–55.54)	1.21	(1.19–1.24)	99.77%
		Curd	2054	35	53.20	(40.1–99.89)	3.58	(3.48–3.69)	99.32%
		Cheese 50	2725	56	96.63	(71.02–165.89)	1.52	(1.47–1.58)	99.05%
		Cheese 110	3563	24	27	(24.5–41.95)	1.41	(1.38–1.45)	99.83%
	Rainy	Milk	6654	26	27.50	(26.15–41.08)	5.21	(5.06–5.36)	99.95%
		Curd	1834	46	63	(51.34–100.16)	2.14	(2.03–2.27)	99.07%
		Cheese 50	4828	81	100.71	(88.32–134.09)	5.12	(4.91–5.34)	99.50%
		Cheese 110	8410	145	291.30	(223.32–418.28)	5	(4.87–5.12)	99.08%
C	Dry	Milk	2388	76	106.67	(87.15–160.35)	4.74	(4.52–4.99)	99.00%
		Curd	7223	77	93.87	(83.15–123.26)	2.44	(2.38–2.50)	99.68%
		Cheese 50	3335	46	69.75	(53.99–116.61)	1.84	(1.78–1.91)	99.4%
		Cheese 110	2180	50	100.14	(68.47–186.09)	1.46	(1.41–1.52)	98.76%
	Rainy	Milk	3589	92	119.60	(102.11–167.35)	9.64	(9.21–10.11)	99.33%
		Curd	2341	41	92	(55.99–214.50)	2.12	(2.03–2.23)	99.23%
		Cheese 50	3386	41	53.75	(45.14–80.28)	1.53	(1.49–1.58)	99.47%
		Cheese 110	3810	26	31.63	(27.32–50.06)	1.18	(1.16–1.2)	99.74%

Higher richness and diversity were observed in samples obtained in the rainy season, particularly from manufacturer A. In general, Chao1 and Inverse Simpson estimators decreased with steps in the cheese process, and following ripening. Despite this, an increase in both estimators was observed during manufacture from manufacturer A and B in the rainy season.

### Diversity of Bacterial Species During Processing

**FIGURE 1:**
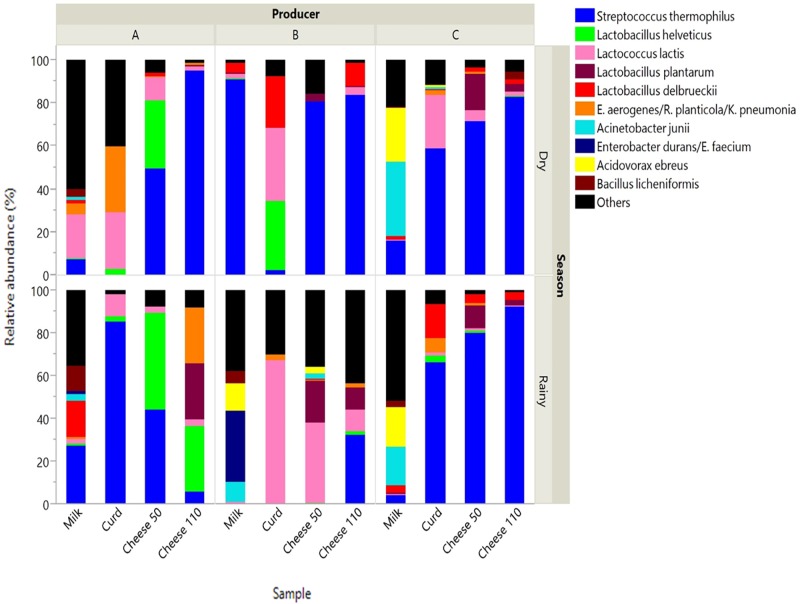
Relative abundance of species in dairy samples collected from Bola de Ocosingo cheese production. Cheese 50: cheese ripened during 50 days. Cheese 110: ripened during 110 days.

Pyrosequencing revealed the bacterial community at species level present in dairy samples collected during Bola de Ocosingo cheese production in two seasons (dry and rainy) among three producers (Figure 1).

Bacterial composition differed across samples type (*P* < 0.05) as main effect (Table 2), in which a reduction in the number of species was observed, some of them becoming predominant at final stages of processing. Also, the interaction between producer and season was statistically significant (*P* < 0.05), in which the predominance of certain species among samples analyzed between producers at the two seasons had an effect.

**Table 2:** PERMANOVA results of Bray-Curtis dissimilarities for main bacteria species found in Bola Cheese production process during two season from three producers.

Terms	Degrees of freedom	Sum of squares	*F*- value by permutation	*R*^2^	*P*
Producer	2	0.5355	2.1919	0.10197	0.077
Season	1	0.2253	1.8443	0.0429	0.118
Sample	3	1.4692	4.0093	0.27978	0.005^∗^
Producer x Season	2	0.7253	2.9687	0.13811	0.04^∗^
Producer x Sample	6	1.0878	1.4842	0.20714	0.187
Season x Sample	3	0.4754	1.2974	0.09053	0.287
Residuals	6	0.7329		0.13957	
Total	23	5.2514		1	

*P-values are based on 999 permutations. ^∗^Statistical significance (P < 0.05).*

In raw milk, the dominant species included bacteria that naturally occur in milk, such as *Streptococcus thermophilus*, *Lactobacillus delbrueckii* and *Lactococcus lactis* (Table 3). *Macrococcus caseolyticus* was found in low abundance, with exception of the sample obtained from manufacturer C in the rainy season, accounting for 22% of reads. Also, environmental microorganisms were observed, including *Acinetobacter junii*, *Bacillus licheniformis*, *Acidovorax* sp. or *Diaphorobacter* sp., and *Elizabethkingia meningoseptica*. For some raw milk samples, there was sporadic occurrence of species known to cause mastitis (i.e., *Enterobacter aerogenes* or *Routella planticola*, *Stenotrophomonas maltophilia*, *Citrobacter freundii*, *Staphylococcus epidermidis* and *L. garviae*).

**Table 3:** Predominant species identified using BLAST from pooled sequences.

Species	% of total reads	Accession number of closest relative	Identity (%)	*E* value
*Streptococcus thermophilus*	44.88	NR 074827.1	98	9E-136
*Lactobacillus helveticus*	11.33	NR 075047.1	99	3E-151
*Lactococcus lactis subsp. lactis*	8.69	NR 103918.1	100	9E-146
*Lactobacillus plantarum*	5.13	NR 075041.1	100	9E-156
*Enterobacter aerogenes/Raoultella planticola*	4.33	NR 102493.1/NR 113701.1	100	2E-141
*Lactobacillus delbrueckii*	2.65	NR 043183.1	100	2E-153
*Acinetobacter junii*	1.74	NR 117623.1	100	4E-139
*Enterococcus durans/E. faecium*	1.56	NR113257.1/NR 114742.1	100	2E-152
*Acidovorax ebreus*	1.45	NR 074591.1	100	5E-138
*Bacillus licheniformis*	1.45	NR 118996.1	100	2E-146
*Lactobacillus futsaii*	1.43	NR 117973.1	99	2E-153
*Enterococcus italicus*	1.13	NR 025625.1	100	3E-150
*Elizabethkingia meningoseptica*	1.06	NR 115236.1	100	4E-139
*Macrococcus caseolyticus*	0.95	NR 074941.1	100	2E-147
*Citrobacter freundii*	0.83	NR 113596.1	100	2E-142
*E.coli/ Shigella flexneri*	0.76	NR 114042.1/NR 026331.1	99	8E-141
*Lactococcus garvieae*	0.69	NR 102968.1	100	7E-147

In curd, *L. lactis* was in all samples (1.54 to 66.96 %), in combination with different LAB, such as *S. thermophilus* (0 to 85.24 %), *L. delbrueckii* (0 to 24.2 %), and/or *L. helveticus* (0 to 32.52 %). Curd sample from manufacturer A in dry season contained a high proportion of *E. aerogenes* or *R. planticola* (30.24 %) and *C*. *freundii* (30.6 %).

During ripening of cheese, species abundances (e.g., *S. thermophilus, L. helveticus, L. delbrueckii, L. lactis* and *L. plantarum*) were different across manufacturers and seasons. For instance, *S. thermophilus* dominated in cheese at 50 days (44.02 to 80.48%) and 110 days (32.37 to 94.67 %) of ripening among all manufacturers in both seasons. However, in cheese at 50 days of ripening made by manufacturer B in the rainy season and in cheese at 110 days made by manufacturer A in the rainy season, there was low abundance of this bacteria (0.023 and 5.43, respectively). For the former, there were other bacteria such as *L. lactis* (37.05 %)*, L. plantarum* (19.64 %) *and L. brevis* (9.61 %), and for the latter, *L. plantarum* (26.04 %), *L. helveticus* (30.88 %) and *E. aerogenes* or *R. planticola* (26.30 %).

## Discussion

This study provides insight into the microbiota present during artisanal production of Bola de Ocosingo cheese from three manufacturers, in dry and rainy seasons. Differences depending of the producer between seasons were observed, in which mainly rainy season samples contained higher diversity. This suggests milk composition and environmental conditions, such as temperature and humidity, affect the types of bacteria introduced during Bola de Ocosingo cheese manufacture. Similar results have been observed during production of other types of cheeses in different seasons (Bonetta et al., 2008a,b; Hinz et al., 2012; Aldrete-Tapia et al., 2014).

Environmental species were observed in raw milk, as well as other bacteria associated with mastitis in cows. A sample from manufacturer C contained high proportions of *M. caseolyticus*, a species in raw milk causing casein breakdown and contributing to formation of aroma precursors (Fuka et al., 2013). All of the species detected have been found in manufacture of other raw milk cheeses, such as Poro (Aldrete-Tapia et al., 2014), Danish (Masoud et al., 2011), Mozzarella (Ercolini et al., 2012), Fontina (Dolci et al., 2014) and Pico cheese (Riquelme et al., 2015), and usually disappear during subsequent processing. This reduction in bacterial diversity is attributed to selection of microorganisms during fermentation and manufacturing processes, attributed to changes in environmental conditions such as pH, moisture, oxygen, water activity, nutrients, and microbial growth inhibitors produced by bacteria or added -externally (Monnet et al., 2014). Yet, as Bola de Ocosingo cheese production includes several processing steps where other raw materials are added by hand, such as adding butter during curd ripening with hand-mixing, and raw milk-derived *pasta filata* used to cover the soft curd and making a round shape cheese. These manufacturing practices represent contamination opportunities, and could explain the presence of other bacteria (e.g., *B. licheniformis*, *E. aerogenes*, *R. planticola*, *Acidovorax* sp.) as observed in manufacturers A and B during the rainy season.

Dominant species in Bola de Ocosingo cheese were LAB, including *S. thermophilus*, *L. delbrueckii*, *L. helveticus* and *L. lactis.* These bacteria are common natural starter cultures, or are inoculated during production of yogurt and Emmental, Gruyere, Parmigiano, Grana, Mozzarella and Cheddar cheese (Bouton et al., 2002; Randazzo et al., 2002; Hols et al., 2005; Delorme, 2008; Aldrete-Tapia et al., 2014). Their principal role is the reduction of pH during manufacture by production of lactic acid (Beresford et al., 2001). Additionally, LAB contribute to aroma and flavor of fermented products (Morales et al., 2003; Leroy and De Vuyst, 2004).

Low proportions of *S. thermophilus* were detected in most raw milk samples. However, during Bola de Ocosingo cheese processing, *S. thermophillus* increased in abundance, while another LAB such as *L. delbrueckii* or *L. helveticus* also increased. This could be due to low molecular weight nitrogen compounds in milk (principally peptides and aminoacids), which are necessary for growth of *S. thermophilus* (Giraffa et al., 2001). Some *S. thermophilus* strains required commensal associations to fulfill nitrogen requirements, possibly provided by the metabolic activity of *L. delbrueckii* and *L. helveticus* (Courtin et al., 2002; Dandoy et al., 2011).

In general, *L. lactis* was present in low levels, but reached higher numbers when *S. thermophilus* was not predominant, for example in curd sample of manufacturer B in rainy season. In this same production, after further processing the proportions of *L. lactis* reduced, overwhelmed by the increase of others bacteria species. This may suggest that *L. lactis* could not dominate during processing, providing an open entrance to other bacteria which could affect quality, even though *L. lactis* is known to rapidly acidify cheese during curd production, preventing proliferation of pathogenic and spoilage species (Wouters et al., 2002; Dandoy et al., 2011).

*Lactobacillus plantarum* was detected in high proportion in some batches, principally during the aging process. This species is a member of the so-called Non-starter LAB (NSLAB) group; NSLAB introduce variability in the ripening process, improving sensory characteristics, but also producing defects (Beresford et al., 2001; Settanni and Moschetti, 2010).

Probiotic effect has been reported for some strains of species detected in bola cheese such as *L. helveticus* (Giraffa, 2014) and potential probiotic properties of *L. delbrueckii*, *S. themophilus* (Mater et al., 2006; Guglielmotti et al., 2007), *L. lactis* (Beck et al., 2015) and *L. plantarum* (Blana et al., 2014).

Interestingly, *Escherichia coli* an indicator of fecal contamination (or the pathogen *Shigella flexneri*) was detected in all samples, with the exception of those from manufacturers A and C during the rainy season. The abundance of this microorganism was very low in raw milk, and remained so until the end of ripening. However, detection of *E. coli* by traditional culture dependent methods was negative in all samples evaluated, which could indicate that cells were dead already, due to changes in physical and chemical parameters during cheese making caused by LAB and the potential antimicrobial compounds generated by their metabolism (Macori and Cotter, 2018). In fact, many LAB isolated from the production of Bola de Ocosingo cheese inhibited the growth of *Salmonella* Typhimurium and *Listeria monocytogenes* in plate wells assays (data not shown), which could possibly validate the production of antimicrobial metabolites, however, more studies should be carried out.

This study provides an insight in microbial community dynamics during Bola de Ocosingo cheese production. *S*. *thermophilus*, *L. lactis*, *L. helveticus*, *L. delbrueckii* and *L. plantarum* dominated during the cheese processing, all reported with potential probiotic effect. Prevalence of these bacteria differed across manufacturers and seasons which could account to differences in final product quality. Pyrosequencing revealed the presence of *E. coli*/*S. flexnerii* in very low proportions even in the ripened cheese. However, detection by traditional methodology was negative. Therefore, to achieve homogeneous cheese quality would be desirable to develop a starter culture by selecting strains with technological characteristics and health benefits, which could be added to pasteurized milk to generate the traditional characteristics of Bola de Ocosingo cheese.

## Author Contributions

AA-T acquired, analyzed,and discussed the data and wrote the manuscript. CE-R did the experimental design, sampled, funded, and revised the draft. MT did the experimental design, funded, analyzed the data, revised the draft, and did the English language editing. MH-I did the experimental design, received funding, revised draft, and wrote the manuscript.

## Conflict of Interest Statement

The authors declare that the research was conducted in the absence of any commercial or financial relationships that could be construed as a potential conflict of interest.

## Footnotes

1 http://www.mrdnalab.com

## Supplementary Material

FIGURE S1 Rarefaction curves of the pyrosequencing reads from dairy samples collected from three producers **(A–C)** of Ocosingo Bola cheese in dry season (continuous line) and rainy season (discontinuous line).
