# Supplementary figures and images for: Characterization of Bacterial Communities in Mexican Artisanal Raw Milk “Bola de Ocosingo” Cheese by High-Throughput Sequencing
Source: Front Microbiol. 2018 Oct 29;9:2598. doi: 10.3389/fmicb.2018.02598 (PMC6217346; doi:10.3389/fmicb.2018.02598)

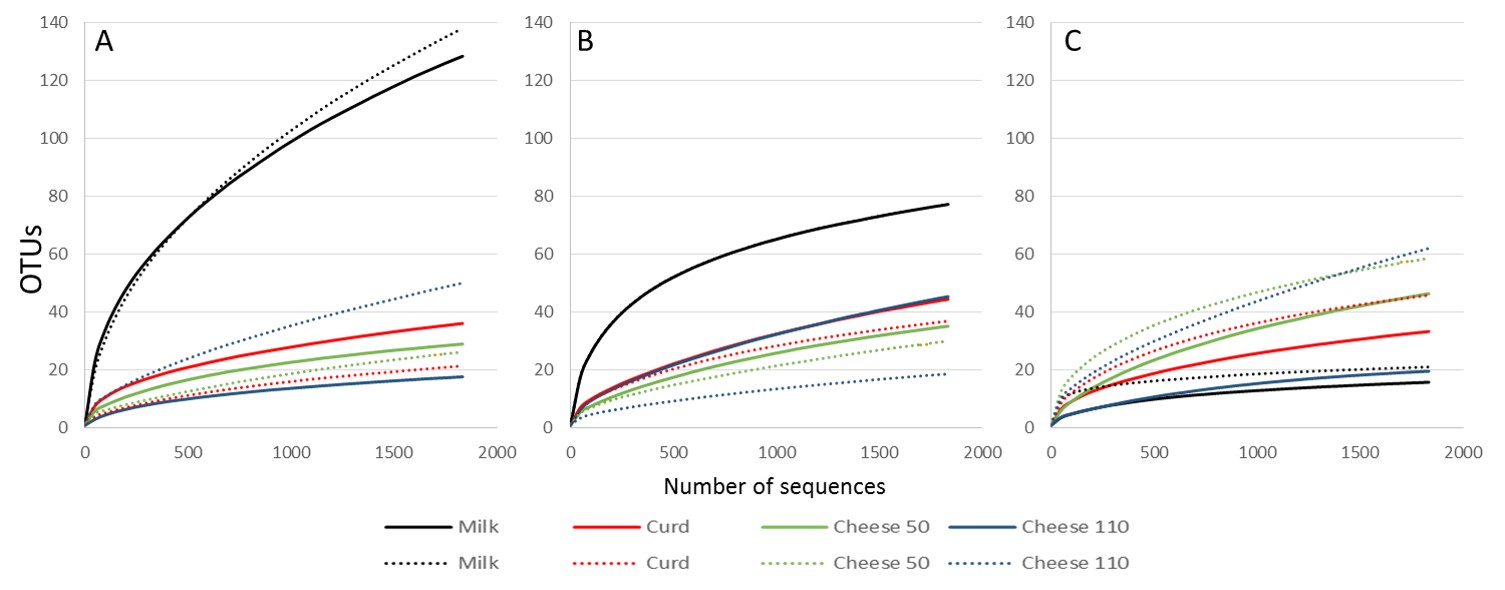

Supplement: FIGURE S1 — Rarefaction curves of the pyrosequencing reads from dairy samples collected from three producers (A–C) of Ocosingo Bola cheese in dry season (continuous line) and rainy season (discontinuous line). [file Image_1.JPEG]
